# Inpatient Opioid Use in Head and Neck Microvascular Free Flap Reconstruction

**DOI:** 10.1002/ohn.70200

**Published:** 2026-03-12

**Authors:** Patricia Timothee, Alessandra Bliss, Daniel Blumenthal, John Dowd, Emily Clementi, Bruce Davidson, Michael Reilly, Matthew Pierce, Jonathan Giurintano

**Affiliations:** ^1^ Department of Otolaryngology–Head & Neck Surgery MedStar Georgetown University Hospital Washington District of Columbia USA; ^2^ Georgetown University School of Medicine Washington District of Columbia USA

**Keywords:** free flap, head neck cancer, opioid, pain

## Abstract

**Objectives:**

The primary objective is to determine whether patients undergoing osseous microvascular free flap reconstruction of head and neck defects have higher inpatient pain requirements than those undergoing nonosseous reconstruction. Secondary aims include evaluating the impact of a multimodal analgesia (MMA) protocol and COVID‐19 visitor restrictions on inpatient opioid administration.

**Study Design:**

Retrospective chart review.

**Setting:**

MedStar Georgetown University Hospital and MedStar Washington Hospital Center.

**Methods:**

Narcotic doses administered during the perioperative inpatient hospitalization were converted to morphine‐equivalent doses (MEDs) for comparison. 2‐tailed *t* tests and *χ* ^2^ analyses were used, with *P* ≤ .05 as the threshold for statistical significance.

**Results:**

318 patients (mean age 64 ± 12.3 years; 65% male) were included. Total inpatient MED was 224.48 and 173.88 in the osseous and nonosseous free flap cohorts, respectively (*P* = .127295). The total MED per day of hospitalization was 16.38 and 17.53, respectively (*P* = .671399). Implementation of an MMA protocol reduced daily MED from an average of 25.9 to 11.24 (*P* < .0001). During COVID‐19 visitor restrictions, total and daily MEDs were 234.57 and 20.64, respectively, compared to 181.32 and 16.9 during unrestricted periods, though these differences were not statistically significant (*P* = .163232 and *P* = .251387, respectively).

**Conclusions:**

The findings suggest no significant difference in postoperative MME between patients undergoing osseous versus non‐osseous free flap reconstruction, and inpatient opioid pain requirements did not differ between groups. MMA protocol implementation was associated with a significant reduction in inpatient narcotic use, while COVID‐19 visitor restrictions had no significant effect.

**Level of Evidence:**

4.

As advances in reconstructive techniques have pushed the limits of what tumors are considered surgically resectable, more patients are undergoing increasingly complex head and neck cancer operations with reconstruction consisting of microvascular free tissue transfer. Depending on the nature of the defect to be reconstructed, microvascular free flaps may transfer a combination of bone, skin, fat, fascia, and muscle from a donor site in the arm, leg, or axilla. The free flaps most commonly used in head and neck reconstruction include the fibula osteocutaneous free flap, scapula osteocutaneous free flap, radial forearm fasciocutaneous free flap, and anterolateral thigh fasciocutaneous free flap. Surgical times for these cases are lengthy, typically between 6 and 12 hours long, as are inpatient hospitalizations, which frequently last longer than one week. Post‐surgical complications are common.^1^

Pain is defined by the International Association for the Study of Pain (IASP) pain as “an unpleasant sensory and emotional experience associated with actual or potential tissue damage or described in terms of such damage.”^2^ Pain consists of both a physiologic and a psychological component, each of which can alter the perceived severity and the level of distress resulting from pain. In head and neck cancer, opioids are typically indicated for moderate to severe pain, regardless of the mechanism, and perioperative pain management in this patient population has traditionally consisted of opioid‐based narcotic medications, using a combination of patient‐controlled analgesia (PCA) opioid infusion, as needed intravenous opioids, and scheduled and/or as needed oral opioids to manage post‐operative pain.^3^, ^4^ While ensuring appropriate pain control is a principle of cancer treatment, the role of prescription opioids in the development of opioid addiction and the rising related to opioid overdose (108,000 overdose deaths in 2022) has led to increased awareness of opioid prescribing patterns and increased emphasis on decreasing opioid prescriptions when able.^5^

In recent years, emphasis has been placed on reducing the necessity for opioid‐based medications in the post‐operative period. Multiple approaches to reducing inpatient and outpatient opioid use have been implemented, including the team‐based enhanced recovery after surgery (ERAS) approach, focused on standardizing perioperative antimicrobial prophylaxis, opioid‐sparing pain regimens, early mobilization, and nutrition plans, and the introduction of opioid‐sparing multimodal analgesia (MMA) protocols, which have been associated with decreased narcotic use and improved postoperative pain management.^6^, ^7^, ^8^, ^9^, ^10^, ^11^ At our institution, an MMA protocol was introduced for all patients undergoing head and neck microvascular reconstruction in 2021. The MMA protocol includes scheduled acetaminophen (650 mg orally), celecoxib (200 mg orally) and gabapentin (300 mg orally), with intravenous ketorolac (15 mg) for breakthrough pain. Intravenous morphine and oral oxycodone are provided as needed for breakthrough pain, and in opioid‐tolerant patients, an opioid PCA (either morphine or hydromorphone) is provided. Additionally, an 81 mg Aspirin is routinely provided to all microvascular free flap patients.

As ERAS and MMA protocols are broadly employed to all patients undergoing head and neck surgery with microvascular free tissue transfer, there remains the potential for overtreatment or undertreatment of individual patients' pain based on factors that have yet to be identified.^12^ For example, microvascular tissue reconstruction may be osseous in nature, using bone harvested from the extremities to reconstruct the craniofacial skeleton, or non‐osseous in nature, using skin, fat, and/or fascia to reconstruct a defect. Similarly, reconstruction may involve the upper aerodigestive tract, necessitating tracheotomy placement to maintain airway patency, or reconstruction may not involve the upper aerodigestive tract and may simply replace external skin or soft tissue. One prior study has shown fibula free flaps to be associated with higher opioid requirements while scapula transfers were associated with decreased opioid requirements compared to other free tissue transfer types; in this study, use of an MMA protocol was paradoxically associated with increased opioid requirements.^13^ There exists limited information regarding the impact of such variables on post‐operative pain requirement in head and neck free flap patients in the literature.

During the recent COVID‐19 pandemic, extreme visitor restrictions were placed on patients undergoing inpatient surgery. At our institution, while elective surgeries were halted for a period of several months during 2020, cancer surgery continued throughout the duration of the pandemic. However, strict visitor precautions were initiated during this time period, with visitors being disallowed from visiting patients undergoing inpatient surgery for almost 30 months. The potential psychosocial impact of these visitor restrictions on post‐surgical pain perception have not been evaluated.

In this study, we seek to retrospectively examine the impact of instituting an MMA protocol for head and neck free flap patients at our institution, we seek to determine if osseous reconstructions have higher inpatient opioid requirement that nonosseous reconstructions, and we seek to examine the impact of COVID‐19 visitor restrictions on postoperative opioid requirements.

## Materials and Methods

After obtaining IRB approval (#00007887) from the MedStar Georgetown University IRB, a retrospective chart review was performed for all patients who underwent microvascular reconstruction by the Otolaryngology–Head and Neck Surgery service at MedStar Georgetown University Hospital and MedStar Washington Hospital Center from January 1, 2018, to June 30, 2024. The study was deemed to be of low risk, and patient consent for medical record review was waived by the MedStar Georgetown University Hospital IRB. Data were deidentified at the point of extraction from the medical record. The primary outcome of the study was total morphine equivalent doses (MED) and MED per day in osseous and non‐osseous free flap patients. Retrospective chart review was performed, recording doses of narcotic medications including morphine, hydromorphone, hydrocodone, oxycodone, fentanyl, methadone, codeine, and tramadol. MED was calculated using conversion factors for each drug (Table 1). Opioid medications given intraoperatively or as part of surgical anesthesia were not included in the calculation of morphine equivalent doses. Additionally, patient‐controlled anesthesia infusions were not calculated as part of the MED as our medical record system does not provide specific or cumulative dose of narcotic provided through this route of administration.

**Table 1:** Opioid MME Conversion Factors

Opioid	Conversion factor
Morphine (mg)	1
Codeine (mg)	0.15
Fentanyl (mcg)	0.1
Hydrocodone	1
Hydromorphone (mg)	4
PO	20
IV	
Oxycodone	1.5
Oxymorphone	3

Secondary outcomes included inpatient MED after initiation of an MMA protocol, and the effect of COVID‐19 visitor restrictions on inpatient MED. Patient characteristics including age, sex, type of free flap, history of radiation therapy, need for tracheotomy at the time of surgery, concurrent neck dissection at the time of surgery, length of operation, and length of hospitalization were all recorded. A 2‐tailed *t* test was used for comparison of parametric data. For categorical variables, *χ* ^2^ testing was performed. All statistical analyses were performed using SPSS statistical software, version 24.0.0.0 (IBM Corp). *P* ≤ .05 was considered to be significant for all statistical analyses.

## Results

From January 1, 2018 until June 30, 2024, 341 head and neck free flap reconstructions were performed. 17 cases were identified as having a history of existing opioid use or abuse, and 6 cases were identified as being cases where initial free tissue transfer failed, and a second free tissue transfer was performed during the same hospitalization; these cases were excluded from the study, with a total of 318 cases included for analysis. The mean age at time of surgery was 64 (range 18‐92, SD 12.3) years. The majority of subjects, n = 206 (64.8%) were males. The majority of flaps performed were soft tissue free flaps, n = 225 (70.8%), and the most commonly performed free flap was the anterolateral thigh flap n = 164 (51.6%) (see Table 2 for patient demographic information).

**Table 2:** Patient Demographics

Characteristic	Value
Sex, No (%)	
Male	206 (65)
Female	112 (35)
Age, mean (range)	64 (18‐92)
Reason for surgery, No (%)	
Cancer	257 (81)
Noncancer	61 (19)
Aerodigestive tract, No (%)	
Yes	258 (81)
No	60 (19)
Time in OR in minutes, mean (range)	666 (271‐1189)
Neck dissection, No. (%)	
Bilateral	80 (25)
Unilateral	128 (40)
None	110 (35)
Tracheotomy, No (%)	
Yes	220 (69)
No	98 (31)
MMA Protocol, No (%)	
Yes	167 (53)
No	151 (47)
PCA Prescription	
Yes	139 (44%)
No	179 (56%)
Days in Hospital, mean (range)	11.4 (1‐121)

The mean opioid requirement for all subjects was 186.7 (SD 274.1) MEDs during the entire postoperative stay. The mean length of stay was 11.4 (range 1‐121, SD 10.1) days, and the average daily dose of opioids was equivalent to 17.0 (SD 22.4) MEDs. Patients who received standardized multimodal anesthesia (MMA) had significantly lower total MED (116.65 vs 293.32, *P* < .0001) and daily MED (11.32 vs 25.9, *P* < .0001) compared to patients who did not receive MMA. In the group receiving MMA, total operative time was noted to be significantly less (620 vs 717 minutes, *P* < .0001), but there were no other significant differences noted (see Table 3 for MMA protocol findings). Patients undergoing osseous free flap reconstruction had significantly longer operative time (738 minutes vs 636 minutes, *P* < .0001), longer duration of hospitalization (13.4 vs 10.5 days, *P* = .02998) and higher rates of tracheotomy (91% vs 59%, *P* < .0001). Despite these differences, there was no difference in total or daily MME between patients undergoing osseous and non‐osseous reconstruction. Of 318 patients, 139 (44%) used a PCA. PCA utilization did not differ significantly across age groups: 18 to 39 years (43%), 40 to 59 years (48%), 60 to 79 years (43%), and 80+ years (36%; *χ*² = 1.46, *P* = .69). However, PCA use was significantly higher among patients undergoing osseous free flaps (55%, *P* = .03) and those not receiving MMA (*P* < .001).

**Table 3:** MMA Protocol Findings

	MMA protocol (n = 167)	No MMA protocol (n = 151)	*P* values
Total MME	116.65	293.32	<.00001
MME/day	11.32	25.9	<.00001
Age, years (mean)	64	63	.9
Operative time, minutes (mean)	620	717	<.00001
Osseous	43 (26%)	50 (32%)	.18
Nonosseous	123 (74%)	103 (68%)	
Neck dissection			
Bilateral	45 (27%)	34 (22%)	.07
Unilateral	73 (44%)	56 (37%)	
None	48 (29%)	63 (20%)	
Tracheotomy			
Yes	115 (69%)	106 (70%)	1
No	51 (31%)	47 (30%)	
History of XRT			
Yes	57 (34%)	64 (42%)	.17
No	109 (66%)	89 (58%)	
OR Takeback			
Yes	21 (13%)	27 (18%)	.21
No	145 (87%)	126 (82%)	
PCA			
Yes	65 (32%)	74 (64%)	<.00001
No	139 (68%)	41 (36%)	

Additional findings demonstrated no significant difference in total mean MED (208.03 vs 172.39, *P* = .45) and daily mean MED (18.73 vs 16.48, *P* = .57) between patients undergoing free flap surgery for oncologic purposes compared to nononcologic purposes. However, patients younger than 65 were found to have significantly higher total MED (276.09 vs 116.65, *P* < .0001) and daily mean MED (21.11 vs 13.27, *P* = .002) compared to patients aged over 65 years.

## Discussion

**Figure 1:**
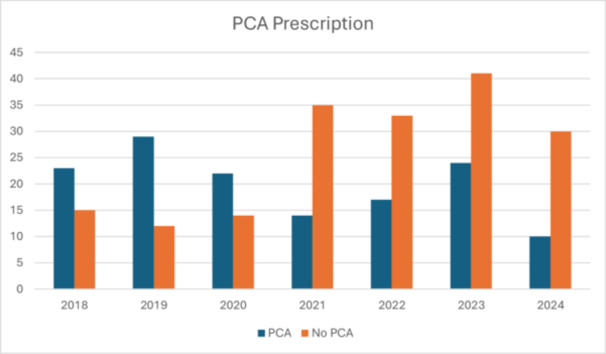
PCA prescriptions from 2018 to 2024.

The impact of MMA on postoperative pain experienced following head and neck surgery with microvascular reconstruction has been mixed, with some studies suggesting that MMA is associated with decreased narcotic use, while others have failed to demonstrate reduction in inpatient opioid prescription.^1^, ^12^ In our large retrospective patient cohort, we demonstrate that since the initiation of a MMA protocol at our institution in 2021, the opioid use of patients undergoing head and neck surgery with microvascular reconstruction has significantly decreased from 25.9 to 11.24 MED/day (*P* < .0001). Additionally, the use of an MMA protocol was associated with significantly decreased use of opioid patient‐controlled analgesia (see Figure 1). These finding provides additional evidence to suggest that MMA is associated with decreased post‐operative opioid use and should be considered across all institutions performing head and neck microvascular reconstruction.

In our analysis, despite osseous free flaps having longer operative time, longer hospital duration, and higher rates of tracheotomy, factors which would be expected to increase pain requirement, there was no difference in postoperative pain requirement between patients undergoing osseous or nonosseous head and neck free flap reconstruction with all other variables, including age, neck dissection, history of radiation therapy, and implementation of MMA protocol, statistically similar. These findings suggest that patients undergoing osseous microvascular reconstruction have similar pain requirements compared to those undergoing soft tissue reconstruction alone, contrary to what has previously been reported in the literature. Additionally, there were no significant differences in inpatient opioid requirements identified between the 4 most common free flaps (see Table 4). There was no significant difference between pain requirements for patients undergoing free flap surgery for oncologic purposes compared to nononcologic purposes (total mean MME 208.03 vs 172.39, *P* = .445871; daily mean MED 18.73 vs 16.48, *P* = .572703). These findings suggest that a standardized analgesia protocol across all patients undergoing head and neck microvascular reconstruction, regardless of flap type or purpose of surgery, is appropriate.

**Table 4:** Osseous Versus Nonosseous Free Flap Findings

	Osseous (n = 93)	Nonosseous (n = 225)	*P* values
Total MME	224.48	172.88	.13
MME/day	16.38	17.56	.67
Age, years (mean)	64	64	1
Operative time, minutes (mean)	738	636	<.00001
Neck dissection			.46
Bilateral	19 (20%)	61 (27%)	
Unilateral	35 (38%)	76 (34%)	
None	39 (42%)	89 (40%)	
Tracheotomy			.00001
Yes	85 (91%)	134 (59%)	
No	8 (9%)	91 (41%)	
History of XRT			.13
Yes	29 (31%)	90 (40%)	
No	64 (69%)	135 (60%)	
Opioid PCA			.03
Yes	49 (53%)	90 (40%)	
No	44 (47%)	135 (60%)	
MMA protocol			.16
Yes	43 (46%)	124 (55%)	
No	50 (54%)	101 (45%)	
OR takeback			.07
Yes	19 (20%)	28 (12%)	
No	74 (80%)	197 (88%)	

A previous study suggested that age over 65 years is associated with the decrease MME requirement in head and neck free flaps. Our data supports these findings. As pain has a psychological component in addition to a physical component, we anticipated that the initiation of strict visitor restrictions during the COVID‐19 pandemic could potentially increase MED requirements, postulating that the absence of social support during inpatient hospitalization could increase pain perception. While patients undergoing head and neck free flap reconstruction during COVID‐19 visitor restrictions trended towards increased inpatient opioid requirements (total mean MED 234.57 vs 181.32, *P* = .16232; daily mean MED 20.64 vs 16.9, *P* = .251387), this finding did not meet a level of statistical significance (see Table 5).

**Table 5:** COVID‐19 Visitor Restriction Findings

	COVID‐19 restrictions (n = 117)	No COVID‐19 restrictions (n = 201)	*P* values
Total MME	211.87	181.32	.37
MME/day	16.9	19.58	.40
Age, years (mean)	64	64	1
Operative time, minutes (mean)	663	668	.98
Neck dissection			
Bilateral	32 (27%)	49 (24%)	.53
Unilateral	35 (30%)	73 (36%)	
None	49 (42%)	79 (39%)	
Tracheotomy			
Yes	86 (65%)	135 (67%)	.28
No	32(35%)	66 (33%)	
History of XRT			
Yes	45 (38%)	75 (37%)	.88
No	73 (62%)	126 (63%)	
Opioid PCA			
Yes	50 (42%)	90 (45%)	.67
No	68 (58%)	111 (55%)	
MMA protocol			
Yes	66 (56%)	101 (50%)	.33
No	52 (44%)	100 (50%)	
OR takeback			
Yes	19 (16%)	27 (13%)	.51
No	99 (84%)	174 (87%)	

Limitations to our study include the retrospective nature of the data and the absence of documentation confirming that pain was adequately controlled in both patient cohorts. While there was no significant difference in opioid requirement between osseous and soft tissue free flaps, it is possible that the pain may be better or worse controlled based on patient experience regardless of the MED administered. Additionally, a potential confounding factor is the administration of an opioid patient‐controlled anesthesia infusion. 44% (n = 139) of patients had an opioid PCA listed in their medication administration record (MAR). Unfortunately, our medical record system does not provide specifics regarding PCA use; there is no record as to whether the PCA was actually used by the patient, the duration of use, or the amount of opioid administered by the PCA. Similarly, the decision to prescribe a PCA is most commonly not made by the Otolaryngology – Head and Neck Surgery team, but is instead made by the Intensive Care Unit team. Patients with a PCA listed on the MAR were found to have significantly higher total and daily MED compared to patients without a PCA (total MED 249.55 vs 137.95, *P* = .00283; daily MED 20.75 vs 14.13, *P* = .008606), suggesting that these patients have a higher baseline pain requirement necessitating use of the PCA. While a significantly greater number of patients undergoing osseous free flap reconstruction had access to a PCA compared to non‐osseous reconstructions (53% vs 40%, *P* = .03486), more focused comparison between subgroups who did and did not receive a PCA shows no significant difference in MMA administration between osseous and nonosseous free flaps in these subgroups (No PCA–total mean MED 138.19 vs 141.02, *P* = .953235; daily mean MED 11.81 vs 15.37, *P* = .392306; Yes PCA–total mean MED 301.95 vs 221.01, *P* = .079297; daily mean MED 20.48 vs 20.89, *P* = .911343). Based on our study analysis comparing the MMA and non‐MMA groups, 32% of the MMA cohort utilized a PCA and had a statically significantly lower overall total and daily narcotic use (both *P* < .00001), suggestive that PCA use is unlikely contributing to the lower MED and MED/day. Age did not significantly influence PCA usage (*P* = .69), though PCA use was more common among patients undergoing osseous free flap reconstruction (55%, *P* = .03) and among non‐MMA patients (*P* < .001). Regardless, the limitations of the medical record system regarding PCA administration limits the extrapolation of the data.

## Conclusions

Institution of a formal MMA protocol at our institution was associated with a significant decrease in post‐operative inpatient opioid use in patients undergoing head and neck free flap reconstruction. Contrary to prior studies, there was no significant difference in postoperative MME between patients undergoing osseous versus non‐osseous free flap reconstruction. Similar to prior reporting, patients aged over 65 years did have significantly lower MME compared to patients under 65 years. Strict COVID‐19 visitor restrictions were not associated with a difference in opioid pain requirement for patients undergoing head and neck free flap reconstruction.

## Author Contributions

**Patricia Timothee**: data collection and analysis, and manuscript development; **Alessandra Bliss** and **Daniel Blumenthal**: data collection and manuscript development; **John Dowd**: data collection and analysis; **Emily Clementi**: manuscript development. **Bruce Davidson**, **Michael Reilly**, and **Matthew Pierce**: manuscript review; **Jonathan Giurintano**: design conception, data collection and analysis, manuscript development, and research supervision.

## Disclosures

### Competing interests

Dr Giurintano receives material research support from Ambu, Inc. The remaining authors have no funding or conflicts of interest to report.

### Funding source

None.
